# Supplementary material for: Bis-Cyclic Guanidine Heterocyclic Peptidomimetics as Opioid Ligands with Mixed μ-, κ- and δ-Opioid Receptor Interactions: A Potential Approach to Novel Analgesics
Source: Int J Mol Sci. 2022 Aug 25;23(17):9623. doi: 10.3390/ijms23179623 (PMC9455983; doi:10.3390/ijms23179623)
Supplement: Supplementary file 1 [file ijms-23-09623-s001.zip › ijms-1885894-supplementary.pdf]

Bis-cyclic guanidine heterocyclic peptidomimetics as opioid ligands with mixed μ-, κ- and δ-opioid receptor interactions: A potential approach to novel analgesics

Jay McLaughlin,[1] Ramanjaneyulu Rayala,[3] Ashley J. Bunnell,[3] Mukund P. Tantak,[3] Shainnel O. Eans,[1] Khadija Nefzi,[1] Michelle L. Ganno,[2] Colette T. Dooley[2] and Adel Nefzi [3*]

[1]Department of Pharmacodynamics, University of Florida, Gainesville, FL 032610, USA.

[2]Torrey Pines Institute for Molecular Studies; 11350 SW Village Parkway; Port St. Lucie, FL 34987, USA.

[3]Center for Translational Science, Florida International University, Port St. Lucie, FL 34987, USA.

Supplementary material:

Invitro screening results against **μ-, κ- and δ** receptors of all compounds.

NMR and MS data for compounds 1968-22, 16, 4 and 1.

In vitro screening results

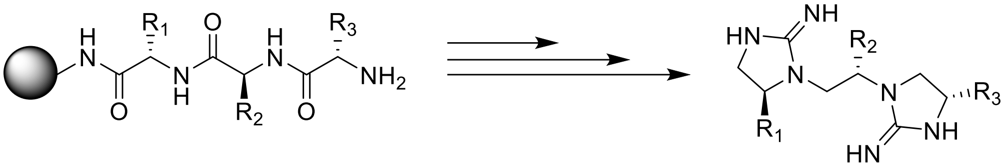

Mu screening results

Sequence of amino acids

1968 #	R1	R2	R3	Ki (Exp 1)	Ki (Exp 2)	MEAN	Ki (nM)	STD
**1**	Phe	Phe	Dmt	3.54E-08	2.49E-08	3.01E-08	30	7.39E-09
2	Phe	Phe	Tyr	1.35E-07	1.42E-07	1.39E-07	139	4.95E-09
3	Phe	Phe	Leu	2.56E-06	2.63E-06	2.59E-06	2591	5.09E-08
**4**	Phe	Tyr	Dmt	1.69E-08	9.51E-09	1.32E-08	13	5.21E-09
5	Phe	Tyr	Phe	9.25E-07	6.71E-07	7.98E-07	798	1.80E-07
6	Phe	Tyr	Tyr	8.45E-08	6.72E-08	7.58E-08	76	1.22E-08
**7**	Tyr	Tyr	Dmt	3.00E-08	3.03E-08	3.01E-08	30	1.91E-10
9	Phe	Leu	Phe	8.48E-07	9.55E-07	9.01E-07	901	7.59E-08
10	Phe	Leu	Tyr	1.99E-07	2.81E-07	2.40E-07	240	5.85E-08
11	Phe	Leu	Leu	2.98E-06	3.04E-06	3.01E-06	3012	3.89E-08
12	Phe	Leu	Dmt	6.74E-08	6.59E-08	6.66E-08	67	1.12E-09
**13**	Ala	Dmt	Phe	1.05E-08	2.12E-08	1.58E-08	16	7.62E-09
14	Phe	Dmt	Tyr	1.35E-07	1.12E-07	1.24E-07	124	1.62E-08
15	Phe	Dmt	Leu	3.50E-07	2.60E-07	3.05E-07	305	6.39E-08
**16**	Ala	Dmt	Tyr	7.52E-09	9.24E-09	8.38E-09	8	1.22E-09
17	Tyr	Phe	Phe	1.07E-06	7.41E-07	9.07E-07	907	2.36E-07
18	Tyr	Phe	Tyr	4.03E-07	4.01E-07	4.02E-07	402	1.48E-09
**19**	Ala	Dmt	Leu	1.59E-08	1.42E-08	1.50E-08	15	1.20E-09
20	Tyr	Phe	Dmt	7.47E-08	8.03E-08	7.75E-08	77	3.97E-09
21	Tyr	Tyr	Phe	1.75E-07	1.99E-07	1.87E-07	187	1.71E-08
**22**	Ala	Dmt	Dmt	7.68E-09	7.31E-09	7.49E-09	7	2.62E-10
23	Tyr	Tyr	Leu	2.35E-06	2.27E-06	2.31E-06	2310	5.59E-08
25	Tyr	Leu	Phe	7.07E-07	7.25E-07	7.16E-07	716	1.32E-08
26	Tyr	Leu	Tyr	7.81E-07	7.51E-07	7.66E-07	766	2.13E-08
27	Tyr	Leu	Leu	9.60E-07	1.00E-06	9.81E-07	981	2.89E-08
28	Tyr	Leu	Dmt	1.18E-07	1.19E-07	1.18E-07	118	9.90E-10
29	Tyr	Dmt	Phe	1.17E-07	1.14E-07	1.16E-07	116	1.70E-09
30	Tyr	Dmt	Tyr	9.88E-08	1.27E-07	1.13E-07	113	1.98E-08
31	Tyr	Dmt	Leu	2.95E-07	2.98E-07	2.97E-07	297	2.62E-09
32	Tyr	Dmt	Dmt	1.06E-07	1.34E-07	1.20E-07	120	1.98E-08
33	Ala	Phe	Phe	4.17E-06	2.54E-06	3.35E-06	3355	1.15E-06
34	Ala	Phe	Tyr	1.48E-06	1.07E-06	1.28E-06	1277	2.88E-07
35	Ala	Phe	Leu	4.45E-06	4.58E-06	4.51E-06	4514	8.91E-08

36	Ala	Phe	Dmt	1.67E-07	1.24E-07	1.46E-07	146	3.05E-08
37	Ala	Tyr	Phe	7.49E-07	5.88E-07	6.69E-07	669	1.14E-07
38	Ala	Tyr	Tyr	1.74E-07	1.95E-07	1.84E-07	184	1.47E-08
39	Ala	Tyr	Leu	1.24E-06	1.77E-06	1.50E-06	1501	3.75E-07
40	Ala	Tyr	Dmt	3.64E-08	4.88E-08	4.26E-08	43	8.79E-09
41	Ala	Leu	Phe	5.62E-06	7.39E-06	6.51E-06	6508	1.25E-06
42	Ala	Leu	Tyr	2.13E-06	2.47E-06	2.30E-06	2300	2.45E-07
43	Ala	Leu	Leu	7.81E-06	1.07E-05	9.26E-06	9260	2.05E-06
44	Ala	Leu	Dmt	1.57E-07	2.44E-07	2.00E-07	200	6.14E-08
49	Dmt	Phe	Phe	5.34E-08	8.47E-08	6.91E-08	69	2.22E-08
50	Dmt	Phe	Tyr	6.16E-08	1.14E-07	8.78E-08	88	3.71E-08
51	Dmt	Phe	Leu	1.26E-07	1.62E-07	1.44E-07	144	2.52E-08
52	Dmt	Phe	Dmt	8.34E-08	1.02E-07	9.28E-08	93	1.33E-08
54	Dmt	Tyr	Tyr	7.23E-08	5.64E-08	6.43E-08	64	1.12E-08
**55**	Dmt	Tyr	Phe	2.74E-08	3.25E-08	2.99E-08	30	3.63E-09
56	Dmt	Tyr	Dmt	5.50E-08	6.99E-08	6.25E-08	62	1.06E-08
57	Dmt	Leu	Phe	5.96E-08	7.41E-08	6.68E-08	67	1.03E-08
58	Dmt	Leu	Tyr	5.51E-08	6.20E-08	5.86E-08	59	4.86E-09
59	Dmt	Leu	Leu	1.66E-07	1.56E-07	1.61E-07	161	6.72E-09
60	Dmt	Leu	Dmt	6.64E-08	7.12E-08	6.88E-08	69	3.38E-09
61	Dmt	Dmt	Phe	4.03E-08	5.65E-08	4.84E-08	48	1.15E-08
62	Dmt	Dmt	Tyr	6.42E-08	7.84E-08	7.13E-08	71	1.00E-08
63	Dmt	Dmt	Leu	6.28E-08	9.88E-08	8.08E-08	81	2.55E-08
64	Dmt	Dmt	Dmt	5.86E-08	8.80E-08	7.33E-08	73	2.08E-08
65	Phe	Phe	Phe	2.37E-06	2.83E-06	2.60E-06	2598	3.29E-07
66	Phe	Tyr	Leu	2.04E-06	1.99E-06	2.02E-06	2017	3.61E-08
67	Phe	Dmt	Phe	2.30E-07	1.77E-07	2.03E-07	203	3.71E-08
68	Phe	Dmt	Dmt	8.93E-08	7.67E-08	8.30E-08	83	8.94E-09
69	Tyr	Phe	Leu	2.08E-07	1.88E-07	1.98E-07	198	1.43E-08
70	Tyr	Tyr	Tyr	2.30E-07	2.82E-07	2.56E-07	256	3.67E-08
71	Dmt	Tyr	Leu	5.80E-08	5.30E-08	5.55E-08	56	3.54E-09

Kappa screening results

1968#	R1	R2	R3	Ki (Exp 1)	Ki (Exp 2)	MEAN	Ki (nM)	STD
1	Phe	Phe	Dmt	7.97E-07	7.29E-07	7.63E-07	763.15	4.82E-08
2	Phe	Phe	Tyr	5.36E-06	5.57E-06	5.46E-06	5461.00	1.47E-07
3	Phe	Phe	Leu	4.51E-06	4.84E-06	4.67E-06	4672.50	2.33E-07
**4**	Phe	Tyr	Dmt	3.11E-07	1.78E-07	2.45E-07	244.60	9.39E-08
5	Phe	Tyr	Phe	3.95E-06	3.73E-06	3.84E-06	3842.50	1.56E-07
6	Phe	Tyr	Tyr	5.35E-06	3.43E-06	4.39E-06	4389.00	1.36E-06
**7**	Tyr	Tyr	Dmt	1.88E-06	9.02E-07	1.39E-06	1388.45	6.88E-07
9	Phe	Leu	Phe	1.14E-05	1.09E-05	1.11E-05	11120.00	3.68E-07
10	Phe	Leu	Tyr	5.47E-06	4.84E-06	5.16E-06	5155.00	4.43E-07
11	Phe	Leu	Leu	1.39E-05	8.93E-06	1.14E-05	11431.50	3.53E-06

12	Phe	Leu	Dmt	1.03E-06	1.29E-06	1.16E-06	1156.00	1.84E-07
**13**	Ala	Dmt	Phe	2.64E-07	2.66E-07	2.65E-07	264.95	1.91E-09
14	Phe	Dmt	Tyr	1.19E-06	1.44E-06	1.31E-06	1314.50	1.82E-07
15	Phe	Dmt	Leu	5.68E-07	5.04E-07	5.36E-07	535.75	4.49E-08
**16**	Ala	Dmt	Tyr	2.46E-07	2.93E-07	2.70E-07	269.60	3.30E-08
17	Tyr	Phe	Phe	6.27E-06	6.84E-06	6.55E-06	6553.00	4.06E-07
18	Tyr	Phe	Tyr	3.47E-06	3.63E-06	3.55E-06	3546.50	1.11E-07
**19**	Ala	Dmt	Leu	8.93E-08	4.38E-08	6.65E-08	66.55	3.22E-08
20	Tyr	Phe	Dmt	9.77E-07	6.59E-07	8.18E-07	818.20	2.25E-07
21	Tyr	Tyr	Phe	3.59E-06	3.29E-06	3.44E-06	3440.00	2.12E-07
**22**	Ala	Dmt	Dmt	1.95E-07	2.42E-07	2.18E-07	218.10	3.32E-08
23	Tyr	Tyr	Leu	9.52E-06	7.36E-06	8.44E-06	8441.50	1.52E-06
25	Tyr	Leu	Phe	6.55E-06	3.32E-06	4.93E-06	4933.00	2.28E-06
26	Tyr	Leu	Tyr	1.58E-05	1.32E-05	1.45E-05	14535.00	1.85E-06
27	Tyr	Leu	Leu	4.24E-06	4.84E-06	4.54E-06	4540.50	4.29E-07
28	Tyr	Leu	Dmt	8.40E-07	1.40E-06	1.12E-06	1117.70	3.92E-07
29	Tyr	Dmt	Phe	1.45E-06	1.45E-06	1.45E-06	1448.50	7.07E-10
30	Tyr	Dmt	Tyr	1.73E-06	1.76E-06	1.74E-06	1744.50	1.77E-08
31	Tyr	Dmt	Leu	2.58E-06	2.08E-06	2.33E-06	2332.00	3.52E-07
32	Tyr	Dmt	Dmt	1.76E-06	1.48E-06	1.62E-06	1618.50	2.03E-07
33	Ala	Phe	Phe	1.63E-05	1.41E-05	1.52E-05	15180.00	1.57E-06
34	Ala	Phe	Tyr	6.52E-06	9.15E-06	7.83E-06	7834.00	1.86E-06
35	Ala	Phe	Leu	3.41E-05	5.26E-05	4.33E-05	43340.00	1.31E-05
36	Ala	Phe	Dmt	1.03E-06	1.32E-06	1.17E-06	1173.50	2.10E-07
37	Ala	Tyr	Phe	3.45E-06	3.88E-06	3.66E-06	3664.00	3.00E-07
38	Ala	Tyr	Tyr	1.69E-06	1.96E-06	1.82E-06	1822.00	1.88E-07
39	Ala	Tyr	Leu	4.09E-06	7.72E-06	5.90E-06	5902.00	2.57E-06
40	Ala	Tyr	Dmt	2.35E-07	2.63E-07	2.49E-07	249.10	2.01E-08
41	Ala	Leu	Phe	2.39E-05	2.20E-05	2.30E-05	22950.00	1.30E-06
42	Ala	Leu	Tyr	1.23E-05	1.23E-05	1.23E-05	12305.00	7.07E-09
43	Ala	Leu	Leu	3.60E-05	5.30E-05	4.45E-05	44480.00	1.20E-05
44	Ala	Leu	Dmt	1.83E-06	1.42E-06	1.63E-06	1625.50	2.89E-07
49	Dmt	Phe	Phe	1.23E-06	1.21E-06	1.22E-06	1223.50	1.48E-08
50	Dmt	Phe	Tyr	1.90E-06	1.74E-06	1.82E-06	1820.00	1.16E-07
51	Dmt	Phe	Leu	8.72E-07	6.39E-07	7.55E-07	755.15	1.65E-07
52	Dmt	Phe	Dmt	1.20E-06	1.24E-06	1.22E-06	1219.50	2.76E-08
54	Dmt	Tyr	Tyr	1.46E-06	1.60E-06	1.53E-06	1528.50	9.69E-08
**55**	Dmt	Tyr	Phe	5.75E-07	5.84E-07	5.79E-07	579.00	6.36E-09
56	Dmt	Tyr	Dmt	7.36E-07	1.25E-06	9.91E-07	991.30	3.62E-07
57	Dmt	Leu	Phe	3.71E-07	6.79E-07	5.25E-07	524.80	2.18E-07
58	Dmt	Leu	Tyr	5.42E-07	7.75E-07	6.59E-07	658.65	1.65E-07
59	Dmt	Leu	Leu	2.23E-07	3.13E-07	2.68E-07	268.05	6.33E-08
60	Dmt	Leu	Dmt	3.08E-07	4.22E-07	3.65E-07	365.25	8.08E-08
61	Dmt	Dmt	Phe	8.77E-07	1.23E-06	1.05E-06	1051.45	2.47E-07
62	Dmt	Dmt	Tyr	2.30E-06	2.34E-06	2.32E-06	2318.50	2.62E-08
63	Dmt	Dmt	Leu	1.64E-06	1.47E-06	1.55E-06	1554.50	1.22E-07

64	Dmt	Dmt	Dmt	2.82E-06	1.59E-06	2.21E-06	2206.00	8.65E-07
65	Phe	Phe	Phe	1.66E-05	1.52E-05	1.59E-05	15910.00	1.03E-06
66	Phe	Tyr	Leu	2.66E-06	3.32E-06	2.99E-06	2990.50	4.69E-07
67	Phe	Dmt	Phe	1.23E-06	1.12E-06	1.17E-06	1170.50	7.85E-08
68	Phe	Dmt	Dmt	5.38E-07	5.68E-07	5.53E-07	552.85	2.17E-08
69	Tyr	Phe	Leu	2.12E-06	2.13E-06	2.12E-06	2124.00	4.24E-09
70	Tyr	Tyr	Tyr	1.11E-05	5.55E-06	8.32E-06	8321.00	3.92E-06
71	Dmt	Tyr	Leu	3.73E-07	3.86E-07	3.80E-07	379.65	8.84E-09

Delta screening results

	AA Sequence			Delta				
1968 #				**Ki**	**Ki**	**MEAN**	**Ki (nM)**	**STD**
**1**	Phe	Phe	Dmt	**5.12E-07**	**5.40E-07**	5.26E-07	526	1.97E-08
2	Phe	Phe	Tyr	6.92E-06	9.28E-06	8.10E-06	8100	1.66E-06
3	Phe	Phe	Leu	**6.37E-06**	**6.95E-06**	6.66E-06	6659	4.04E-07
**4**	Phe	Tyr	Dmt	7.29E-07	1.02E-06	8.74E-07	874	2.06E-07
5	Phe	Tyr	Phe	6.40E-06	1.21E-05	9.23E-06	9227	3.99E-06
6	Phe	Tyr	Tyr	**4.88E-06**	**5.09E-06**	4.98E-06	4983	1.47E-07
**7**	Tyr	Tyr	Dmt	2.00E-06	1.99E-06	2.00E-06	1997	7.78E-09
9	Phe	Leu	Phe	9.83E-06	**5.81E-06**	7.82E-06	7819	2.85E-06
10	Phe	Leu	Tyr	2.23E-06	2.19E-06	2.21E-06	2208	2.55E-08
11	Phe	Leu	Leu	2.64E-05	9.09E-05	5.86E-05	58630	4.56E-05
12	Phe	Leu	Dmt	1.95E-06	1.75E-06	1.85E-06	1850	1.36E-07
**13**	Ala	Dmt	Phe	7.41E-07	8.55E-07	7.98E-07	798	8.08E-08
14	Phe	Dmt	Tyr	1.00E-06	1.12E-06	1.06E-06	1062	8.63E-08
15	Phe	Dmt	Leu	1.07E-06	1.44E-06	1.25E-06	1254	2.60E-07
**16**	Ala	Dmt	Tyr	6.16E-07	7.77E-07	6.97E-07	697	1.14E-07
17	Tyr	Phe	Phe	7.93E-06	6.27E-06	7.10E-06	7100	1.17E-06
18	Tyr	Phe	Tyr	7.57E-06	8.58E-06	8.08E-06	8076	7.16E-07
**19**	Ala	Dmt	Leu	1.18E-06	1.01E-06	1.09E-06	1095	1.25E-07
20	Tyr	Phe	Dmt	8.77E-07	8.29E-07	8.53E-07	853	3.39E-08
21	Tyr	Tyr	Phe	7.83E-06	7.63E-06	7.73E-06	7732	1.41E-07
**22**	Ala	Dmt	Dmt	2.60E-07	2.71E-07	2.65E-07	265	7.57E-09
23	Tyr	Tyr	Leu	1.58E-04	**3.22E-02**	1.62E-02	16154100	2.26E-02
25	Tyr	Leu	Phe	2.54E-05	3.57E-05	3.05E-05	30515	7.30E-06
26	Tyr	Leu	Tyr	7.48E-06	8.67E-06	8.07E-06	8074	8.43E-07
27	Tyr	Leu	Leu	1.75E-04	**4.66E+05**	2.33E+05	########	3.30E+05
28	Tyr	Leu	Dmt	4.80E-06	5.34E-06	5.07E-06	5069	3.86E-07
29	Tyr	Dmt	Phe	9.67E-07	7.05E-07	8.36E-07	836	1.85E-07
30	Tyr	Dmt	Tyr	1.34E-06	1.07E-06	1.20E-06	1203	1.87E-07
31	Tyr	Dmt	Leu	2.20E-06	1.68E-06	1.94E-06	1940	3.66E-07
32	Tyr	Dmt	Dmt	**4.69E-07**	5.07E-07	4.88E-07	488	2.70E-08

33	Ala	Phe	Phe	1.66E-05	**5.95E-06**	1.13E-05	11263	7.52E-06
34	Ala	Phe	Tyr	**5.14E-06**	8.16E-06	6.65E-06	6650	2.14E-06
35	Ala	Phe	Leu	9.43E-05	**1.79E+08**	8.93E+07	########	1.26E+08
36	Ala	Phe	Dmt	3.71E-06	3.40E-06	3.55E-06	3551	2.21E-07
37	Ala	Tyr	Phe	3.43E-06	3.80E-06	3.61E-06	3614	2.67E-07
38	Ala	Tyr	Tyr	**4.27E-06**	3.33E-06	3.80E-06	3800	6.63E-07
39	Ala	Tyr	Leu	4.15E-06	3.51E-06	3.83E-06	3833	4.50E-07
40	Ala	Tyr	Dmt	1.52E-06	1.32E-06	1.42E-06	1415	1.41E-07
41	Ala	Leu	Phe	**7.31E-06**	**1.54E+00**	7.71E-01	########	1.09E+00
42	Ala	Leu	Tyr	2.06E-05	9.08E-05	5.57E-05	55670	4.96E-05
43	Ala	Leu	Leu	**9.25E-06**	**6.69E-06**	7.97E-06	7971	1.81E-06
44	Ala	Leu	Dmt	1.02E-05	**5.06E-06**	7.63E-06	7628	3.64E-06
49	Dmt	Phe	Phe	6.15E-07	**4.72E-07**	5.43E-07	543	1.01E-07
50	Dmt	Phe	Tyr	1.10E-06	6.90E-07	8.96E-07	896	2.90E-07
51	Dmt	Phe	Leu	2.58E-06	2.53E-06	2.55E-06	2555	4.17E-08
52	Dmt	Phe	Dmt	5.82E-07	**5.21E-07**	5.51E-07	551	4.31E-08
54	Dmt	Tyr	Tyr	3.55E-06	3.01E-06	3.28E-06	3279	3.87E-07
**55**	Dmt	Tyr	Phe	1.24E-06	1.20E-06	1.22E-06	1219	2.76E-08
56	Dmt	Tyr	Dmt	1.33E-06	1.26E-06	1.30E-06	1296	4.60E-08
57	Dmt	Leu	Phe	1.40E-06	1.07E-06	1.24E-06	1238	2.33E-07
58	Dmt	Leu	Tyr	1.15E-06	6.38E-07	8.96E-07	896	3.64E-07
59	Dmt	Leu	Leu	3.84E-06	4.58E-06	4.21E-06	4211	5.28E-07
60	Dmt	Leu	Dmt	9.78E-07	6.51E-07	8.14E-07	814	2.32E-07
61	Dmt	Dmt	Phe	1.06E-06	9.85E-07	1.02E-06	1020	4.94E-08
62	Dmt	Dmt	Tyr	1.53E-06	1.02E-06	1.28E-06	1276	3.61E-07
63	Dmt	Dmt	Leu	2.27E-06	2.51E-06	2.39E-06	2390	1.70E-07
64	Dmt	Dmt	Dmt	**5.08E-07**	6.50E-07	5.79E-07	579	1.01E-07
65	Phe	Phe	Phe	7.34E-06	7.29E-06	7.32E-06	7317	3.25E-08
66	Phe	Tyr	Leu	1.82E-05	**5.76E+10**	2.88E+10	########	4.07E+10
67	Phe	Dmt	Phe	8.28E-07	8.33E-07	8.30E-07	830	3.68E-09
68	Phe	Dmt	Dmt	4.26E-07	4.98E-07	4.62E-07	462	5.11E-08
69	Tyr	Phe	Leu	4.23E-06	5.02E-06	4.63E-06	4629	5.59E-07
70	Tyr	Tyr	Tyr	9.96E-06	1.17E-05	1.08E-05	10820	1.22E-06
71	Dmt	Tyr	Leu	3.83E-06	3.02E-06	3.43E-06	3427	5.76E-07

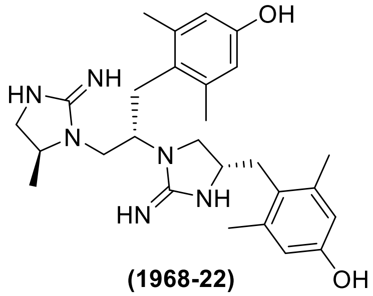

(1968-22)

Chemical Formula: $C_{27}H_{38}N_6O_2$
Exact Mass: 478.31
Molecular Weight: 478.64

^{1}H NMR (400 MHz, DMSO-d_6) δ 10.38 (s, 1H), 9.80 (s, 1H), 8.82 (s, 1H), 8.38 (s, 3H), 6.47 (s, 2H), 6.42 (s, 2H), 4.20 – 4.11 (m, 1H), 4.08 – 4.01 (m, 1H), 4.00 – 3.93 (m, 2H), 3.88 (t, *J* = 9.5 Hz, 1H), 3.50 (t, *J* = 9.3 Hz, 1H), 3.17 (dd, *J* = 9.8, 4.2 Hz, 1H), 3.04 – 2.93 (m, 1H), 2.84 – 2.71 (m, 4H), 2.62 (dd, *J* = 14.4, 4.7 Hz, 1H), 2.25 (s, 6H), 2.15 (s, 6H), 0.97 (d, *J* = 6.2 Hz, 3H).

^{13}C NMR (100 MHz, DMSO-d_6) δ 158.40, 157.97, 155.61, 155.39, 137.71, 137.68, 123.86, 123.82, 115.23, 115.06, 52.48, 52.33, 47.27, 33.95, 28.57, 20.3, 20.1, 16.69.

MS: 479.6 (MH+)

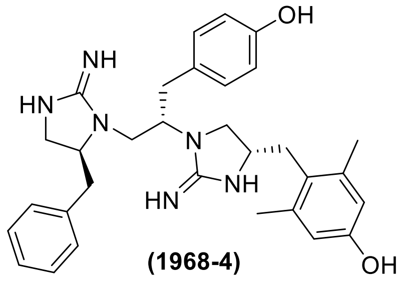

(1968-4)

Chemical Formula: $C_{31}H_{38}N_6O_2$
Exact Mass: 526.31
Molecular Weight: 526.69

^{1}H NMR (400 MHz, DMSO-d_6) δ 10.08 (s, 1H), 9.03 (s, 2H), 8.37 (s, 3H), 7.31 (ddd, *J* = 19.5, 13.5, 5.9 Hz, 6H), 7.17 (d, *J* = 8.5 Hz, 2H), 6.73 (d, *J* = 8.5 Hz, 2H), 6.40 (s, 1H), 4.53 – 4.39 (m, 2H), 4.01 – 3.88 (m, 2H), 3.72 (t, *J* = 9.6 Hz, 1H), 3.53 (s, 1H), 3.46 – 3.36 (m, 2H), 3.35 – 3.29 (m, 1H), 3.01 (dd, *J* = 13.5, 3.8 Hz, 1H), 2.83 (dd, *J* = 14.4, 4.7 Hz, 1H), 2.76 – 2.65 (m, 3H), 2.56 (dd, *J* = 14.3, 4.0 Hz, 1H), 2.12 (s, 6H).

^{13}C NMR (100 MHz, DMSO-d_6) δ 158.15, 156.24, 155.36, 137.74, 136.03, 129.75, 129.48, 128.47, 126.76, 126.34, 123.91, 115.32, 115.01, 57.27, 52.32, 44.45, 41.63, 35.87, 34.07, 33.68, 20.07.

MS: (527.7 (MH+)

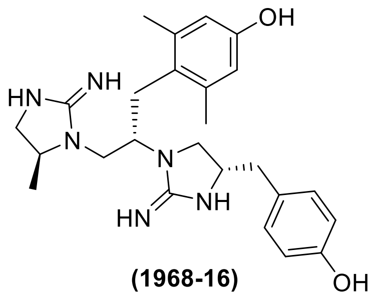

(1968-16)

Chemical Formula: $C_{25}H_{34}N_6O_2$
Exact Mass: 450.27
Molecular Weight: 450.59

^{1}H NMR (400 MHz, DMSO-d_6) δ 8.39 (s, 2H), 6.92 (d, *J* = 8.5 Hz, 2H), 6.69 (d, *J* = 8.5 Hz, 2H), 6.44 (s, 2H), 6.32 (t, *J* = 5.4 Hz, 1H), 5.66 (s, 2H), 4.05 – 3.87 (m, 2H), 3.41 (t, *J* = 9.2 Hz, 1H), 3.29 – 3.18 (m, 3H), 3.02 – 2.93 (m, 3H), 2.89 (dd, *J* = 14.3, 7.0 Hz, 1H), 2.76 (dd, *J* = 14.8, 3.3 Hz, 1H), 2.61 (dd, *J* = 14.2, 8.0 Hz, 1H), 2.37 (d, *J* = 6.8 Hz, 2H), 2.16 (s, 6H), 1.06 (d, *J* = 6.7 Hz, 3H).

^{13}C NMR (100 MHz, DMSO-d_6) δ 158.80, 157.23, 155.79, 155.57, 137.68, 129.76, 128.52, 123.28, 115.24, 115.19, 55.21, 50.65, 49.77, 45.91, 41.96, 40.6, 30.78, 20.12, 14.6.

MS: 451.6 (MH+)

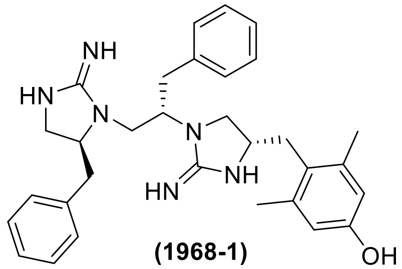

(1968-1)

Chemical Formula: $C_{31}H_{38}N_6O$
Exact Mass: 510.31
Molecular Weight: 510.69

^{1}H NMR (400 MHz, DMSO-d_6) δ 9.61 (s, 4H), 8.36 (s, 1H), 7.42 – 7.23 (m, 10H), 6.38 (s, 2H), 4.58 (s, 1H), 4.49 – 4.38 (m, 1H), 4.00 (dd, *J* = 15.2, 10.4 Hz, 1H), 3.88 (ddd, *J* = 14.1, 9.4, 4.8 Hz, 1H), 3.70 (t, *J* = 9.5 Hz, 1H), 3.53 (s, 1H), 3.40 – 3.24 (m, 3H), 3.02 (dd, *J* = 13.5, 4.0 Hz, 1H), 2.96 (dd, *J* = 14.5, 5.1 Hz, 1H), 2.82 (dd, *J* = 14.2, 9.8 Hz, 1H), 2.75 – 2.65 (m, 2H), 2.55 (dd, *J* = 14.3, 4.0 Hz, 1H), 2.11 (s, 6H).
MS: 511.7 (M+)
